# Patient-Derived Nasopharyngeal Cancer Organoids for Disease Modeling and Radiation Dose Optimization

**DOI:** 10.3389/fonc.2021.622244

**Published:** 2021-02-23

**Authors:** Sasidharan Swarnalatha Lucky, Martin Law, Ming Hong Lui, Jamie Mong, Junli Shi, Sidney Yu, Do Kun Yoon, Shih Kien Djeng, Jiguang Wang, Chwee Ming Lim, Min Han Tan

**Affiliations:** ^1^ Institute of Bioengineering and Nanotechnology, Agency for Science Technology and Research (A*STAR), Singapore, Singapore; ^2^ Proton Therapy Centre Pte Ltd., Singapore, Singapore; ^3^ Department of Chemical and Biological Engineering, The Hong Kong University of Science and Technology, Hong Kong, Hong Kong; ^4^ Division of Life Science, Department of Chemical and Biological Engineering, Center for Systems Biology and Human Health and State Key Laboratory of Molecular Neuroscience, The Hong Kong University of Science and Technology, Hong Kong, Hong Kong; ^5^ Department of Otorhinolaryngology-Head and Neck Surgery, Singapore General Hospital, Singapore, Singapore; ^6^ Department of Otolaryngology, National University Health System, Yong Loo Lin School of Medicine, National University of Singapore, Singapore, Singapore

**Keywords:** recurrent NPC, organoids model, radioresistance, oxygen enhancement ratio, hypoxia, linear quadratic model, patient-derived xenografts, radiotherapy

## Abstract

Effective radiation treatment (RT) for recurrent nasopharyngeal cancers (NPC), featuring an intrinsic hypoxic sub-volume, remains a clinical challenge. Lack of disease‐specific *in-vitro* models of NPC, together with difficulties in establishing patient derived xenograft (PDX) models, have further hindered development of personalized therapeutic options. Herein, we established two NPC organoid lines from recurrent NPC PDX models and further characterized and compared these models with original patient tumors using RNA sequencing analysis. Organoids were cultured in hypoxic conditions to examine the effects of hypoxia and radioresistance. These models were then utilized to determine the radiobiological parameters, such as α/β ratio and oxygen enhancement ratio (OER), characteristic to radiosensitive normoxic and radioresistant hypoxic NPC, using simple dose-survival data analytic tools. The results were further validated *in-vitro* and *in-vivo*, to determine the optimal boost dose and fractionation regimen required to achieve effective NPC tumor regression. Despite the differences in tumor microenvironment due to the lack of human stroma, RNA sequencing analysis revealed good correlation of NPC PDX and organoid models with patient tumors. Additionally, the established models also mimicked inter-tumoral heterogeneity. Hypoxic NPC organoids were highly radioresistant and had high α/β ratio compared to its normoxic counterparts. *In-vitro* and *in-vivo* fractionation studies showed that hypoxic NPC was less sensitive to RT fractionation scheme and required a large bolus dose or 1.4 times of the fractionated dose that was effective against normoxic cells in order to compensate for oxygen deficiency. This study is the first direct experimental evidence to predict optimal RT boost dose required to cause sufficient damage to recurrent hypoxic NPC tumor cells, which can be further used to develop dose-painting algorithms in clinical practice.

## Introduction

Nasopharyngeal carcinoma (NPC) is endemic in the east and southeast Asia, where 95% of the cases are invariably associated with Epstein-Barr virus (EBV) infection (1). Radiotherapy (RT) has been the mainstay treatment for early-stage NPC. However >50% of the patients present with locally advanced and distant metastasis during initial diagnosis, reducing the 5-year survival rates to 50–70% (2). Local recurrences are observed in ≈10% of the patients following initial RT, representing a substantial challenge to oncologists (3, 4). Re-treatment with RT (dose ≥60 Gy) is employed in 70–80% of inoperable advanced recurrent cases (5), often resulting in late complications (6–8), further reducing the 5-year survival to ≤50% (9–11). Hence, there is an urgent need for optimized treatment combination and personalized RT planning to achieve local tumor control without significant late morbidities in advanced recurrent NPC.

With significant advancements in diagnostic technology, suboptimal doses and marginal misses may not be the principal cause for local failure. In fact, most locoregional recurrences occurred in the high dose region of the gross tumor volume (GTV) (12), suggesting a strong biological relationship between clonal selection and proliferation of radioresistant cells at the primary site (13). This could be linked to the intrinsic radioresistant hypoxic environment of recurrent NPC, which in turn compromises radiation induced cellular damage and apoptosis; resulting in angiogenesis, tumor progression, and radioresistance (14).

Prevention of recurrence due to hypoxic cell survival may require a higher radiation dose to hypoxic sub-volumes to compensate for oxygen insufficiency. Nevertheless, it remains unclear how much boost dose is required to eliminate hypoxic NPC cells and whether they are sensitive to dose fractionation. Although, the α/β ratio of NPC tissue is generally assumed to be 10 Gy (15), there is no experimental evidence determining the radiobiological parameters specific to NPC tissues or the optimal dose escalation required to eliminate radioresistant cells in the hypoxic sub-volume.

Here in, we established *in-vitro* hypoxic organoids models, mimicking the hypoxic radioresistant sub-volumes of recurrent NPC. We then employed simple and straightforward radiation dose-survival data analytic techniques that yields quantitative readouts defining the inherent radiobiological parameters, such as α/β ratio and oxygen enhancement ratio (OER) of radiosensitive normoxic and radioresistant hypoxic NPC. We then validated the effectiveness of the experimentally calculated RT boost dose in controlling the growth of *in-vitro* and *in-vivo* patient derived NPC tumor models to determine an optimal boost dose and fractionation regimen to obliterate the radioresistant hypoxic cells.

## Materials and Methods

### NPC Patient Participants and Samples

Eighteen NPC tissue samples were obtained from patients who underwent biopsy or surgical resection at the National University Hospital Singapore between March 2015 and April 2019. Specimen collection and experimental use were approved by the Institutional Review Board of National Healthcare Group (DSRB Reference: 2015/00098-SRF0004). One part of the tissue collected from patients were immediately transferred to RPMI-1460 media with HEPES and L-Glutamine and 5X antibiotic/antimycotic and 5 µg/ml Metronidazole at 4°C. Other part was fixed in 10% neutral buffered formalin (10% NBF) for routine Hematoxylin and Eosin (H&E) staining and remaining tissues were snap frozen in liquid nitrogen for DNA and RNA extraction.

### NPC PDX Implantation

NPC patient sample was cut into 4–5 mm pieces and immersed in 1:1 Geltrex : HBSS (Gibco). For subcutaneous implantation, 2–3 mm nick was made on the skin of an anaesthetized NSG mice to insert the explanted tissue. For renal capsular implantation, an incision was made in the mouse kidney to insert the tumor tissue in the subcapsular space. All animal care and experimental procedures were approved by the Institutional Animal Care and Use Committee, A*Star Research Entities, Singapore.

### Establishment of Organoid Cultures

**Figure 1:**
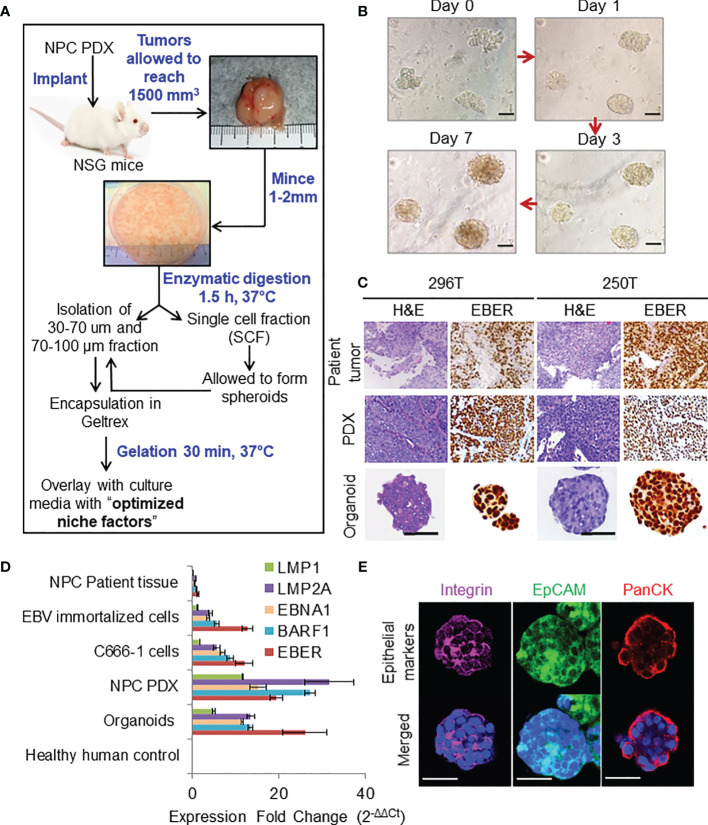
Organoid establishment and characterization. **(A)** Schematic of organoid culture and establishment. **(B)** Organoid growth pattern and morphology; magnification 200×, scale bar 100 µm. **(C)** Representative images of H&E staining and corresponding EBER RNA ISH of 296T and 250T patient tumors and corresponding PDXs as well as organoids; magnification 400×, scale bar 100 µm. **(D)** Expression of latent EBV genes in different NPC samples quantified by qPCR, fold changes are relative to healthy human control samples and normalized by changes in beta-actin values, n = 3. **(E)** Representative confocal microscopy images of organoids following immunofluorescent staining with anti-integrin (magenta), anti-EpCAM (green), and anti-Pan cytokeratin (PanCK) (red) antibodies. Lower panel is the merged image with nuclear staining using Hoechst 33342 (blue); magnification 600×, scale bar 100 µm.

A modified cancer tissue-originated spheroid (CTOS) method was adopted for establishment of NPC organoids from xenograft tumors (**Figure 1A**) (16). Briefly, PDX tissues were manually minced into 1–2 mm^3^ pieces and subjected to enzymatic digestion using 0.28 U/ml Liberase DH (Roche) in RPMI-1460 containing 5% Penicillin-Streptomycin, 20 mM HEPES, 5 U/ml DNase1, and 30 µM Y-27632 (Stemcell Technologies) for 1.5 h at 37°C. The tissue digest was then resuspended in ice-cold HBSS with 5 U/ml DNase1 and 30 µM Y-27632. To remove large undigested pieces, the cell suspension was filtered through 100 µm cell strainer, followed by isolation of the 70–100 µm and 30–70 µm fractions using 70 µm and 30 µm MACS Smart Strainer (Miltenyi Biotech) respectively. Organoids were first counted and then gently mixed with Geltrex (Gibco) at 1.2% (w/v). Fifty microliters of the organoid suspension was seeded in 96-well ultra-low attachment plates (Corning). The gel matrix was then overlaid with 200 µl of complete PneumaCult™-ALI (P-ALI) media (Stemcell Technologies) supplemented with niche factors (NF): 4 µg/ml Heparin, 125 ng/ml Hydrocortisone, 50 ng/ml EGF, 50 ng/ml bFGF, 20 ng/ml FGF-10, 10 µM SB 202190, 500 nM A83-01, 10 µM Y-27632, and 2 mM Glutamax.

### C666-1 Cell Monolayer and Spheroids Culture

C666-1 cells (RRID : CVCL_7949) were authenticated using STR (Short tandem repeat) profiling and were free of mycoplasma contamination. The cells were cultured in RPMI‐1640 medium supplemented with 10% FBS at 37°C in a humidified incubator under 5% CO_2_. Confluent cells (70–80%) were sub-passaged by incubation with 0.05% trypsin for 5 min at 37°C and a splitting ratio of 1:3 was used. For monolayer cultures of C666-1 in 96-well high binding flat bottom plates, cells were seeded at a concentration of 5,000 cells/well. For spheroid cultures, cells were seeded in 96-well flat bottomed ultra-low attachment plates at a concentration of 8,000 cells/well.

### RNA Sequencing and Analysis

Approximately 1 μg RNA per sample (patient biopsy, PDX tumors, organoids, and cell-line) was used to construct the complementary (cDNA) library. Briefly, for all the patient samples, total RNA samples extracted from tissues, except for one patient RNA sample (250T) RNA was extracted from formalin fixed paraffin embedded (FFPE) tissue. Ribosomal RNA (rRNA) was removed from total RNA using Ribo-zero™ rRNA Removal Kit (Epicentre, Madison, WI, USA). For the other RNA samples from PDX tissues, organoid, and cells, rRNA depletion was not carried out. Strand-specific RNA-seq libraries were prepared using the NEBNext^®^ Ultra™ Directional RNA Library Prep Kit for Illumina^®^ (NEB, UK), as per manufacturer’s instructions. Sequencing was performed on an Illumina^®^ with NovaSeq 6000 (Illumina, USA) to generate 150 bp paired-end reads. For patient samples, coding and long non-coding RNA was sequenced, whereas only the coding mRNA was sequenced for all other samples.

Raw.fastq sequencing results are separated into human and mouse reads using Xenome with hg19 human genome and GRCm38 p6 mouse genome. Reads mapped exclusively to human are aligned to Gencode hg19 transcriptome using STAR and quantified by RSEM to obtain FPKM (Fragments Per Kilobase per Million mapped reads) and raw counts per gene for downstream analyses. Only the protein coding genes (n = 20,330) were considered.

### Differential Gene Expression and Functional Enrichment

Gene ontology (GO) analysis was performed with Gene Set Enrichment Analysis (GSEA) when inferring from the whole protein-coding transcriptome, or with g:Profiler when inferring from a subset of labeled genes. We separately performed GO analysis using the GSEA Hallmark gene set, Kyoto Encyclopedia of Genes and Genomes (KEGG) annotations, ontology of biological processes (GO : BP), and cellular components (GO : CC).

For GSEA, the input matrix was the read counts per gene calculated from RSEM, which was then normalized by size factor estimation from DESeq2 on a per-sample basis. Due to the limited number of available samples, false-discovery rate was controlled by permuting the gene sets instead of phenotypes. Ontologies with a family-wise error rate (FWER) <0.05 were deemed significantly different between phenotypes.

Principal component analysis (PCA) was used in discerning the inter-sample differences. Principal components were calculated from the top 1,000 most dispersed protein-coding genes, measured by the log mean-variance ratio (logVMR) of the log-FPKM expression values from RSEM. The ontology of genes contributing to the top principal components (PC) are further considered using g:Profiler. The analysis was separated by the positive and negative contributions to the PC, and ontology were deemed significantly different with adjusted p-value <0.05.

### *In-Vitro* Single Dose RT

All irradiation was performed using Gammacell with ^137^Cs source (Nordion, Canada) at a dose rate of 1 Gy/min with ±15% error. To determine the radiobiological parameters of NPC, C666-1 monolayer and spheroids cultures as well as organoids, were irradiated once at doses ranging from 0.2 to 30 Gy on Day 5. Cell viability was measured on days 14 and 21 using the RealTime-Glo™ MT Cell Viability Assay and percentage of viable cells were expressed relative to that in the untreated well.

### Cell Viability Assays

C666-1 cell, C666-1 spheroid, and NPC organoid viability was determined by RealTime-Glo™ MT Cell Viability Assay (Promega) following manufacturer’s protocol. Briefly, old culture media was replaced with 200 µl of respective culture media containing MT Cell Viability Substrate and NanoLuc^®^ Enzyme at 1X concentration. For monolayer culture, the luminescence measurements could be taken from 1 h following the addition of the reagents but for 3D cultures a minimum of 6 h incubation was required. Luminescence values of the treated group were normalized to respective control non-irradiated organoids grown in normoxic and hypoxic conditions. This ATP independent cell-viability assay was employed to determine the dose-response survival following irradiation of the cells/organoids. A complementary CellTiter-Glo^®^ 3D cell viability assay was used as an end-point assay for 3D cultures following manufacturer’s protocol with some modification. Briefly, 50 µl of 5 U/ml Dispase (Stemcell Technologies) was added to each well to digest the gel and the plate was incubated at 37°C for 45 min. One hundred microliters of Cell-Titre glo 3D reagent was added and incubated for another 1 h at 37°C before measuring the luminescence using a plate reader.

A Live/dead cell imaging assay was also done to qualitatively determine the number of live and dead cells using Live/Dead^®^ viability/cytotoxicity kit (Molecular Probes) following manufacturer’s protocol. Briefly, 2 μM calcein AM and 4 μM Ethidium Homodimer-1 in sterile, tissue culture grade D-PBS were added to the well directly and incubated for 1 h before imaging using a fluorescent microscope with standard FITC and TRITC filters. Samples were washed two more times with HBSS before mounting on eight-well chambered cover glass slide (µ-Slide 8 Well, ibidi^®^).

### *In-Vivo* RT

Fifty NOD Scid Gamma (NSG) mice (5–6 weeks old, 22 ± 2 g) were subcutaneously implanted with NPC PDX tissues at the left flank. When the tumors reached an average size of about 200 mm^3^, the mice were randomly assigned to six groups (seven mice per group) (i) Control (20% v/v DMSO in saline); (ii) Chemotherapy (CT) alone—5 mg/kg Cisplatin (Cis) and 100 mg/kg 5-Fluorouracil (5-FU) dissolved in DMSO and diluted 20% v/v in saline—i.p. administration on days 1 and 7; (iii) eight doses of 2 Gy; (iv) eight doses of 2 Gy + CT on days 1 and 7; (v) two doses of 8 Gy; and (vi) two doses of 8 Gy + CT on days 1 and 7. Tumor volume was measured every other day, at least 3 days a week, using the formula:

(1) $$\text{Tumor volume}=\left(\text{length}\times {\text{width}}^{2}\right)/2$$

### Statistical Analysis

Statistical analyses were performed by two-way ANOVA with Tukey’s multiple comparisons test using GraphPad Prism (Version 8, USA). Curve fitting of the linear quadratic (LQ) model were performed using SPSS (IBM, Version 25, USA). Values of α and β were derived from the best fit to the survival curves of normoxic and hypoxic condition, from which the oxygen enhancement ratio (OER) was then calculated using the formula (17):

(2) $$\text{OER}=\frac{\frac{\alpha }{\beta }\;(\text{hypoxia})}{\frac{\alpha }{\beta }\;(\text{normoxia})}$$

Detailed experimental information is available in the **Supplementary Materials and Methods**.

## Results

### Establishment and Characterization of NPC PDXs

Of 18 NPC patient samples (10 newly diagnosed and 8 recurrent) implanted in NSG mice [mostly subcutaneous (SC) at the flank or both sub-renal (SR) and SC], only five proceeded to passage 1 (P1), showing modest transplantable success rate of 27.8%. Two were lost due to bacterial infections at P2 and P4, and the third could not be maintained beyond P2. The remaining two PDX lines [296T (SC) and 250T (SR)] took about 2.5 and 4 months respectively to be passaged from P0 to P1. While 296T xenografts were propagated every 2 months after P6 and 250T had an average time to propagation of ≈4 months. In an effort to maintain these lines, we revived the slow frozen samples at P3-P5 and achieved stable PDX growth following SC implantation with ≈60% success rate. The detailed clinical information of all 18 donors are listed in **Table 1**.

**Table 1:** Clinical data of donor NPC patients.

	Tissue Source	Recurrent or newly diagnosed	Clinical stage	Pathology diagnosis	EBV positive	Treatment details	PDX	RNA- seq
1	Primary (250T)	Recurrent	T4N0M1	NPC	Yes	Palliative CT	Yes	Yes
2	Lymph node (296T)	Recurrent	T0N1M0	NPC	Yes	Surgery	Yes	Yes
3	Primary	New	T4N0M0	NPC	Yes	ChemoRT		
4	Primary	New	T2N1M0	NPC	Yes	ChemoRT		
5	Primary	New	T3N2M0	NPC	Yes	ChemoRT		
6	Primary	New	T4N0M0	NPC	Yes	Induction CT + ChemoRT	Up to P4	
7	Primary	New	T4N1M0	NPC	Yes	Induction Chemo + ChemoRT	Up to P2	
9	Lymph node	Recurrent	T0N1M0	NPC	Yes	Surgery and CT		
9	Primary	New	T2N2M1	NPC	Yes	Palliative CT		Yes
10	Primary	New	T2N1M1	NPC	Yes	Palliative CT		
11	Primary	New	T3N0M1	NPC	Yes	Palliative CT		Yes
12	Primary	Recurrent	T1N2M1	NPC	Yes	Palliative CT		
13	Primary	Recurrent	T4N2M0	NPC	Yes	ChemoRT		
14	Primary	New	T1N0M0	NPC	Yes	RT		
15	Primary	Recurrent	T4N0M0	NPC	Yes	Re-RT	Up to P1	Yes
16	Lymph node	New	T0N2M0	NPC	Yes	ChemoRT		Yes
17	Primary	Recurrent	T1N2M1	NPC	Yes	Palliative CT		Yes
18	Primary	Recurrent	T1N0M0	NPC	Yes	Surgery		

### Establishment and Characterization of NPC Organoids From PDXs

Representative images of organoid growth and morphology are shown in **Figure 1B**. Organoids were grown in various commercially available media to fine tune the optimal growth and viability (**Figures S1A, B**). P-ALI media with optimized NF supported organoid viability for up to 45 days (**Figures S1C, D**). Organoids could also be cultured from single cell fraction (SCF) that self-assembled to form spheroids (**Figures S1E, F**), which were then encapsulated in Geltrex (**Figure S1G**) or re-implanted in animals to establish xenograft tumors (**Figure S1H**) with similar histopathological features and EBV expression as that of the respective PDX (**Figure S1I, J**). Histologically, patient tumors, corresponding PDXs, and organoids featured atypical cells with high nuclear/cytoplasmic ratio (**Figure 1C**). Presence of EBV was confirmed by RNA *in-situ* hybridization (ISH) for Epstein–Barr virus-encoded small RNA (EBER) (**Figure 1C**). QPCR further confirmed that organoids expressed latent EBV genes (**Figure 1D**), as well as displayed distinct cell borders [integrin and epithelial cell-adhesion molecule (EpCAM) staining] and cytoplasmic keratinization (pan-cytokeratin staining), which are histologic characteristics of squamous cell carcinoma (**Figure 1E**). Organoids were also positive for multifunctional stem cell marker and cell-adhesion glycoprotein CD44 (**Figures S2A, B**), however they did not exhibit expression of other cancer stem cell markers such as OCT4, NANOG, SOX2, or ALDH1. qPCR analysis of immediate early (BZLF-1 and BRLF-1) and late (BLLF-1) lytic genes revealed significantly higher expression of BZLF-1 genes in the early passages of PDX and early phase of organoid growth (**Figure S2C**), as reported previously (18).

### Transcriptomic Fidelity of PDX and Organoids

**Figure 2:**
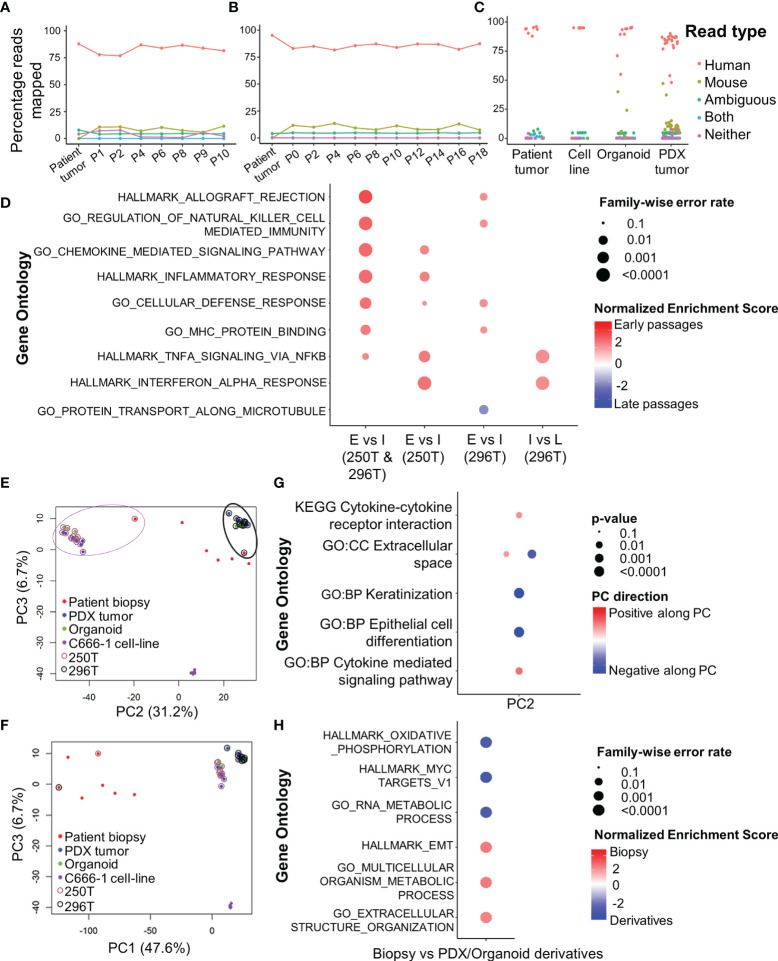
RNA-seq analysis of primary human tumors, and corresponding PDX and organoid models. Xenome output mapping of **(A)** 250T patient tumor and corresponding PDX tissue at different passages ranging from P0-P10, **(B)** 296T patient tumor and corresponding PDX tissue at different passages ranging from P1-P18, and **(C)** different samples including patient tumor biopsies, C666-1 cell line (monolayer and spheroids), PDXs, and organoids. **(D)** Top gene ontologies based on gene expressions between the different passages of the PDX. E, Early passages (≤P2); I, intermediate passages (P3-P10); and L, late passages (>P10, only applicable to 296T). **(E, F)** PCA showing the clustering of primary tumors (biopsies), PDXs, organoids, and c666-1 cell line samples. PC1 corresponds to heterogeneity between the primary tumors, PDXs, as well as organoids; PC2 corresponds to the inter-tumoral differences; PC3 corresponds to the differences between C666-1 cell-lines and primary tumors and its derivatives (PDX and organoids). **(G)** Top different gene ontologies inferred from the top genes contributing to PC2. **(H)** Top different gene ontologies based on gene expressions between the original tumor and PDX/organoids.

RNA-seq analysis of both PDX and organoids revealed a high percentage of reads uniquely mapped to human reference genome (average 82 and 87.1%, **Figures 2A–C**). Comparison between early (≤P2), intermediate (P3-P10), and late passages (P10-P18) using GSEA showed significantly up-regulated expression of chemokines and inflammatory pathways in earlier passages of the PDX compared to the intermediate and late passages (**Figure 2D**). PCA (**Figures 2E, F**) showed the existence of inter-tumoral differences between the various biopsies and PDX/organoid derivatives along PC2, where the patient tumors were most dispersed. However, the established models (250T and 296T) were well-clustered within the same patient group, while being far apart from each other. The top 100 contributing genes on PC2 suggested the relative differences lied in the extracellular space, keratinization, and epithelial cell differentiation based on g:Profiler analysis (**Figure 2G**). On comparing the gene expression of the biopsies with the PDX/organoids derivatives using GSEA, we observed significant up-regulation in metabolism, together with extracellular matrix (ECM) organization and epithelial-mesenchymal transition (EMT) in biopsies, while there was a significant down-regulation of genes related to ribosomal proteins, MYC targets, and oxidative phosphorylation in the derivatives (**Figure 2H**).

### Establishment of Hypoxic Radioresistant NPC Organoids

**Figure 3:**
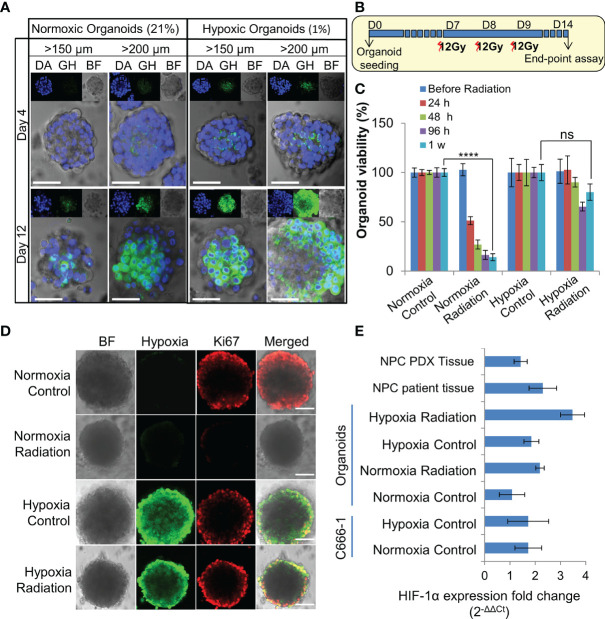
Establishment and characterization of radioresistant hypoxic organoids. **(A)** Representative confocal microscopic images of normoxic (21% oxygen) and hypoxic (1% oxygen) organoids of various sizes stained by Green Hypoxia Reagent (GHR), top panel shows nuclear staining by DAPI (blue), hypoxia staining by GHR (green), and bright field imaging (gray scale); magnification 400×, Scale bar 100 µm. **(B)** Schematic of high-dose radiation regimen to establish radioresistant organoids. **(C)** Relative cell viability of normoxic and hypoxic organoids relative to respective control untreated organoids at day 14 (D14), n = 3, ns, not significant, ****p < 0.0001. **(D)** Representative confocal microscopic images of normoxic and hypoxic organoids without and with radiation (5Gy) showing proliferation of cells (anti-Ki67 staining-red) in the periphery; magnification 400×, hypoxia staining by GHR (green), BF, Bright field image, Scale bar 100 µm. **(E)** Expression of HIF-1α in different NPC samples quantified by RT-PCR, fold-changes are relative to normoxic control organoids and normalized to changes in the GAPDH gene expression, n = 3.

Organoids were cultured in 1% hypoxic incubator to establish *in-vitro* hypoxic NPC model. Staining with Green Hypoxia Reagent (GHR) revealed significant hypoxic areas in hypoxic organoids beyond 200 µm in diameter as early as day 4 (**Figures 3A** and **S3A–B**). Irradiation of normoxic organoids with a dose of 12 Gy on 3 consecutive days (**Figure 3B**) led to about 85% reduction in cell viability by 1 week (**Figure 3C**). In sharp contrast, hypoxic organoids revealed radioresistance and had no significant reduction in end-point cell viability. Staining the organoids with anti-Ki-67 following RT revealed active ≈2-fold proliferation of the cells in the peripheral region of the hypoxic organoids, but not in the normoxic organoids (**Figures 3D** and **S3C**). QPCR analysis indicated twice the expression of hypoxia inducible factor-1α (HIF-1α) in non-irradiated hypoxic organoids compared to normoxic counterparts, and further ≈2 folds increase following RT (**Figure 3E**). Furthermore, there was a significant γ-H2AX phosphorylation, corresponding to DNA double-strand breaks in normoxic organoids following RT (**Figure S3D**).

### Organoid Models as a Platform to Establish Radiobiological Parameters for NPC

**Figure 4:**
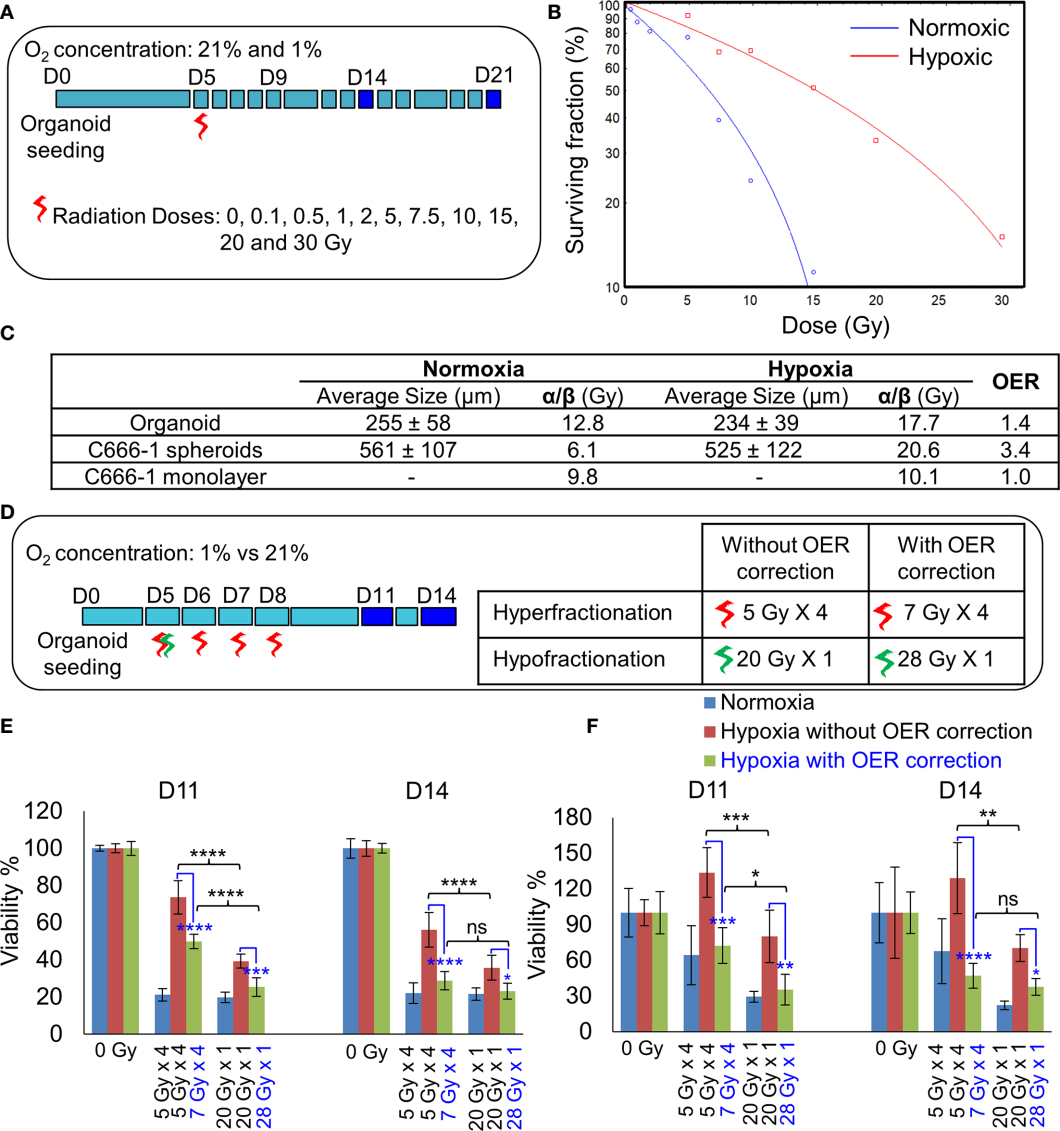
Establishment of radiobiological parameters of NPC. **(A)** Schematic of RT regimen for establishment of α/β ratio, dark blue box represents days on which data was used to plot the LQ curve. **(B)** Representative radiation dose-survival (LQ) curves of organoids in normoxic and hypoxic conditions at D21. **(C)** Tabulated summary of the average size of the organoids/spheroids at Day 14, experimentally calculated α/β ratio of various NPC models and their OER values. **(D)** Schematic of *in-vitro* RT regimen to validate experimentally established OER. Viability of 296T **(E)** and 250T organoids **(F)** following single bolus and fractionated RT regimen without and with OER correction, n = 5. ns, not significant, *p = 0.05, **p = 0.005, ***p < 0.001, ****p < 0.0001. OER, oxygen enhancement ratio.

The differences in the radiobiological characteristics and treatment outcomes of normoxic and hypoxic organoids suggested that they have distinct radiobiological parameters. While re-RT could control the proliferation of normoxic areas within recurrent NPC, effective control of radioresistant hypoxic areas might require a larger RT dose. In order to determine the boost in RT dose required by the hypoxic sub-volume, we irradiated organoids, C666-1 spheroids as well as monolayer cultures just once, at doses ranging from 0.5 to 30 Gy (**Figure 4A**). Changes in cell survival as a function of the dose at day 21 are illustrated (**Figure 4B**), to determine radiobiological parameters α, β (**Table 2**) and α/β ratio (**Figure 4C**). Hypoxic NPC organoids displayed a higher α/β ratio than that of normoxic ones. The OER, which is the boost in RT dose required to achieve similar biological effect in hypoxic cells as compared to normoxic cells, was found to be about 1.4 for organoids Normoxic and hypoxic C666-1 monolayer had similar α/β ratio. However, hypoxic C666-1 spheroids showed ≈3 times higher α/β ratio compared to normoxic spheroids. Owing to their self-assembly in low-attachment surfaces, spheroids had a larger and non-uniform size compared to organoids, perhaps resulting in a larger hypoxic core when cultured at hypoxic conditions, and thus a higher OER (**Figure 4C**).

**Table 2:** Radiobiological parameters of normoxic (21% oxygen concentration) and hypoxic (1% oxygen concentration) organoids, C666-1 spheroids, and monolayer, derived from fitting survival data Days 14 and 21 in LQ model.

NORMOXIA (21%)							
Day	Cell type	α (Gy^−1^)	Standard error of α	β (Gy^−2^)	Standard error of β	α/β (Gy)	Goodness of fit
D14	Organoids	0.0131	0.0057	0.0014	0.0002	9.3494	0.9869
	C666-1 spheroids	0.0206	0.0095	0.0033	0.0008	6.1500	0.9820
	C666-1 monolayer	0.0552	0.0258	0.0064	0.0020	8.6202	0.9886
D21	Organoids	0.0873	0.0105	0.0054	0.0012	16.2259	0.9991
	C666-1 spheroids	0.0368	0.0093	0.0061	0.0007	6.0730	0.9960
	C666-1 monolayer	0.0956	0.0349	0.0087	0.0041	10.9675	0.9850
**HYPOXIA (1%)**							
**Day**	**Cell type**	**α (Gy^−1^)**	**Standard error of α**	**β (Gy^−2^)**	**Standard error of β**	**α/β (Gy)**	**Goodness of fit**
D14	Organoids	0.0460	0.0090	0.0029	0.0010	15.6889	0.9974
	C666-1 spheroids	0.0349	0.0201	0.0030	0.0016	11.7556	0.9681
	C666-1 monolayer	0.0216	0.0098	0.0017	0.0004	12.5394	0.9791
D21	Organoids	0.0254	0.0097	0.0013	0.0004	19.6918	0.9740
	C666-1 spheroids	0.0615	0.0190	0.0021	0.0011	29.3534	0.9880
	C666-1 monolayer	0.0323	0.0184	0.0042	0.0015	7.7581	0.9725

### Hypoxic Organoids Are Less Sensitive to Hyperfractionated RT

On comparing the effect of RT without and with fractionation (**Figure 4D**), we found that a single large dose caused significant reduction in cell viability compared to smaller fractionated doses in hypoxic organoids (**Figures 4E, F**). OER correction further improved the cell killing efficiency of the fractionated dose and resulted in the same amount of cell death in hypoxic organoids as in the normoxic organoids. Hence, hypoxic cells require a large bolus radiation dose, or 1.4 times of the fractionated dose that is effective against normoxic cells.

### Hypofractionated RT Results in Substantial Tumor Growth Delay *In-Vivo*

**Figure 5:**
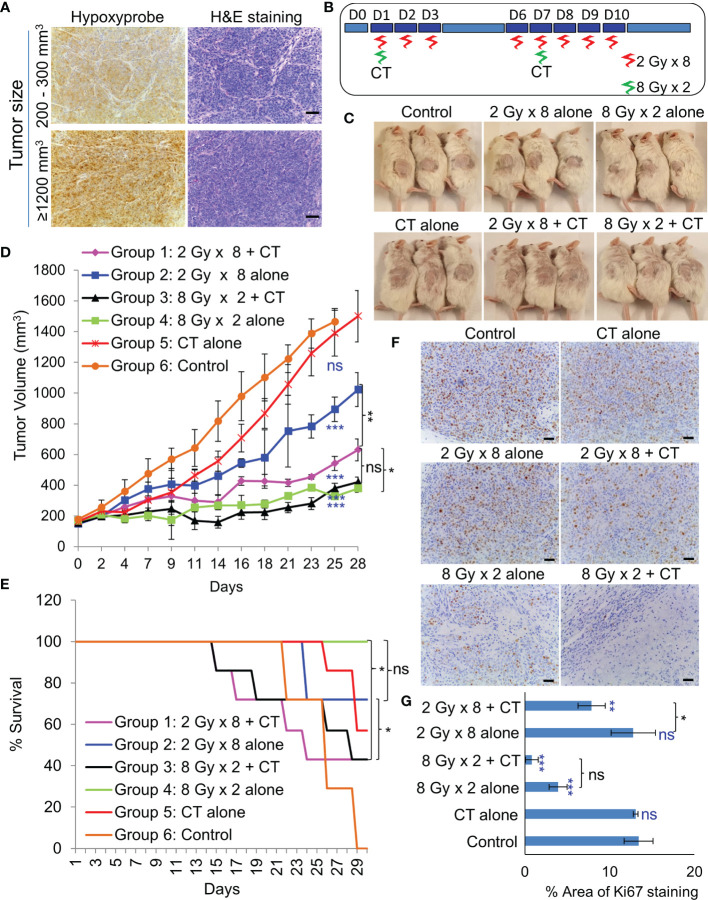
Effect of *in-vivo* RT. **(A)** Representative images of immunohistochemical staining of hypoxic regions using Hypoxyprobe and corresponding H&E staining of tumor tissue; magnification 200×, scale bar 200 µm. **(B)** Schematic of RT regimen with and without CT. **(C)** Representative images of mice showing the size of PDX (296T) tumors at Day 20 from the start of the treatment. **(D)** Tumor growth volume measurements from day 0 to 28, n = 7, ns, not significant, *p = 0.0065, **p = 0.0001, ***p < 0.0001. Blue asterisk denotes p values of treatment group *vs.* untreated control at day 25. **(E)** Survival curve following the treatment of the animals up to day 30, n = 7. Statistical analysis was performed using the log-rank test, *p < 0.05. **(F)** Representative images of immunohistochemical staining of Ki67 in the harvested tumor tissue following different treatment, magnification 200×, scale bar 250 µm. **(G)** Percent area of Ki-67 positive nuclear staining in harvested tumor tissues, n = 3, *p < 0.05, **p < 0.01, ***p < 0.0001. Blue asterisk denotes p values of treatment group *vs.* untreated control.

As NSG mice are extremely sensitive to RT due the PRKDC gene mutation (19), the maximum tolerable whole body irradiation dose is 4 Gy. Targeted irradiation using a custom-made lead shield to irradiate the tumor-site alone, allowed escalation of the dose up to 8 Gy/week. Before irradiation, extend of hypoxia within different sized tumors were analyzed. Even a 200–300 mm^3^ PDX tumor had considerably large hypoxic areas (**Figure 5A**). *In-vivo* treatment (**Figure 5B**) results revealed that RT with two 8 Gy doses led to significant tumor growth control with 100% survival (**Figures 5C–E**). While, the addition of CT to this group did not further improve tumor control, it resulted in 50% drop in survival rate by day 29. Addition of CT alongside fractionated RT (2Gy × 8), on the other hand, displayed significant tumor growth control compared to RT alone. However, there was still no survival benefit due to the severe toxicity associated with CT. Besides toxicity, the animals in this group also suffered from paralysis of the left-hind leg, which was the site of irradiation. Staining of the harvested tumors with proliferation marker Ki67, revealed far lesser number of proliferative tumor cells following radiation with 8 Gy × 2 compared to 2 Gy × 8 dose (**Figures 5F, G**).

### Single *vs* Combined Treatment Modalities

Standard biologically effective dose (BED) model states that biological effect of a dose (d) per fraction (f) given to a tissue in n fractions is given by:

(3) BED=nd [1+d/(α/β)]

where BED is expressed in Gy_α/β_ (20). The standard RT dose scheme has been prescribed as a total given dose of 60 Gy in 2 Gy/f, resulting in total BED of 72 Gy_10_. Conventionally, the prescribed dose for hyper- and hypo-fractionated RT is 1.8 and 4 Gy respectively (17). Taking into account the experimentally determined OER value of 1.4, we propose 2.52 Gy/f for hyperfractionated RT and 5.6 Gy/f for hypofractionated RT. **Table 3** summarizes different RT schemes and their calculated number of fractions for each scheme based on their corresponding BED_α/β_ being close to the standard dose scheme of 72 Gy_10_.

**Table 3:** Tabulation of the proposed photon dose scheme and its calculated BED_α/β_ based on experimental values of α/β ratios and of oxygen enhancement ratio (OER) [Eqn. (3)].

Dose schemes	Hypoxic condition		
	Physical dose	Number of	BED_α/β_
	per fraction (Gy)	fractions	(Gy)
1. Conventional photon	2	30	72
2. Hyperfractionated photon	2.52	25	72
3. Hypofractionated photon	5.6	10	74

Values of α/β = 10 Gy and OER = 1 were used in the conventional photon dose scheme (1), while α/β = 17.7 Gy and OER = 1.4 were applied to hyperfractionated (2) and hypofractionated (3) dose scheme. BED_10_ = 72 Gy is referred as the standard conventional radiotherapy dose scheme in the current study. Physical dose per fraction in hyperfractionated scheme of 1.8 Gy*1.4 = 2.52 Gy and in hypofractionated scheme of 4 Gy*1.4 = 5.6 Gy with α/β = 17.7 Gy were used to calculate the number of fractions with their corresponding BED_α/β_ close to 72 Gy to match the dose scheme with the standard reference dose scheme.

## Discussion

Locoregional recurrences in NPC patients may potentially arise from “in-field” radioresistant hypoxic cancer cells that survived the previous course of treatment (21). Hence, more aggressive RT may be required to eliminate these cells, which is practically impossible due to the adverse side effects of re-treatment and the inability to precisely target the GTV while sparing the surrounding critical structures. Lack of accurate models to optimize and personalize re-treatment regimens due to the difficulty in establishing *in-vitro* and *in-vivo* patient derived NPC models (22), is another obstacle impeding research progress in the field. For decades, attempts to establish *in vitro* EBV-positive NPC cell lines have been disappointing, either with the disappearance of all epithelial cells due to the outgrowth of fibroblasts or the emergence of an EBV-negative epithelial cell line after long-term cultures (23). In fact, this lack of EBV was not just due to the loss of the EBV episome, but there is evidence of widespread HeLa contamination in several NPC cell lines such as CNE1, CNE2, AdAH, NPC-KT, and HONE1 (24, 25). Traditionally, NPC cells were cultured by passaging the tumors as xenografts in immunocompromised mice, such as the widely studied C15 tumor, which retains EBV (26). The only NPC cell-line consistently harboring EBV is C666-1 cells, derived from Xeno-666 (NPC xenograft derived from an undifferentiated patient tumor) (22) and is currently the gold standard used in NPC research. Recently, two more NPC cell-line models carrying EBV were established, which definitely are invaluable tools in NPC research (18, 22).

In the present study, we established two PDX lines and further utilized them to establish *in-vitro* 3D models of hypoxic radioresistant NPC for the first time. Unlike other cancer types, NPC biopsy specimens are tiny and insufficient for direct organoid establishment. RNA-seq analysis revealed good correlation between biopsies and corresponding PDXs and organoids, suggesting the suitability of PDX tissues as a sustainable source for organoid establishment. As reported previously (18, 22, 27), take rate of PDX-engraftment was very modest and the two successful lines were both obtained from recurrent NPC patients. Lin et al. reported the possibility of reactivation of lytic EBV during the transplantation of human tissues into immune-suppressed mice (18). Early lytic genes have been detected in a small fraction of NPC patient tumors (28), but a significant upregulation of BZLF-1 in our early PDX and organoids suggests a clonal selection of a sub-population of cells with an abortive lytic reactivation of EBV in early phases of the cultures (29). This may have also resulted in the observed upregulation of host immune and inflammatory reactions, perhaps resulting in tumor cell death and reduced take rate in mice. On the other hand, establishment of organoids from PDX was 100% successful. Biologically, the major difference between the patient samples and PDX/organoids may lie in the tumor microenvironment. Strict ECM regulation may be lost during engraftment of the tumor tissue in murine host, and further replacement of human stroma with murine derived ECM may have contributed to the downregulation of pathways associated with ECM, EMT, and metabolic processes in PDX and organoids. NPC being a lymphoepithelial tumor, there is a strong dependency of immune-cell and stroma-rich tumor microenvironment on its growth and proliferation (22). Hence, the lack of human stroma is the major drawback of our model as RT also affects the tumor microenvironment, besides the cancer cells. Yet, 3D culture systems closely mimic cell-cell interactions, cellular heterogeneity induced by variations in diffusion of oxygen, growth factors and nutrients from the outer layer to the core, and hence represent realistic proliferation rates compared to 2D cultures.

Here, we developed hypoxic NPC organoid model to study the radioresistance of the hypoxic sub-volumes in recurrent radioresistant NPC. We chose 1% oxygen concentration or physiological hypoxia (30) for *in-vitro* experiments as it is widely used in the literature. Secondly HIF-1α, a major regulator of transcriptional responses to hypoxia, stabilizes at that concentration (31). Standard colony forming assays used to evaluate 2D cell proliferation and survival, however was not applicable for organoid cultures as there was no significant change in the organoid size following treatment, despite a significant cell death especially within the first 10–14 days. Previously, ATP-based end-point luminescence assays was determined to be the best available option to evaluate viability of 3D cultures (32). ATP-assay quantifies mitochondrial activity and indirectly reflect the viable cell numbers. However, radiation induced mitochondrial biogenesis and hyperactivation of mitochondria, may result in inaccurate estimation of viable cells (33). The method we used in this study, measures the reducing potential of viable cells, hence is an ATP-independent method and has the added advantage of continuously monitoring viability.

Irradiation of NPC organoids revealed that the hypoxic organoids that mimicked the radioresistant hypoxic sub-volume, required multiple high RT dose before responding to therapy, while the normoxic counterpart seemed to be susceptible to RT. The observed radioresistance was due to the activation of HIF-1α that could trigger multiple downstream signaling pathways leading to proliferation and survival of hypoxic NPC cells (34). The observed differences in radiobiological characteristics and treatment outcomes between normoxic and hypoxic organoids suggest that they have distinct radiobiological parameters. It is interesting to note that hypoxic organoids had higher α/β ratio, indicating that they might be less sensitive to fractionation (35). Whereas, C666-1 monolayer displayed α/β ratio close to the assumed value of 10 Gy. Our *in-vitro* data comparing fractionated dose with bolus radiation dose, revealed that a modest boost of 40% of the original dose to the normoxic fraction is sufficient to cause significant cell damage to the hypoxic sub-volumes, and these cells were less sensitive to fractionation. As the PDXs were established from patients who failed initial chemoRT with Cisplatin and Gemcitabine, we added another combination of CT drugs to evaluate its efficacy *in-vivo*. Although there was some benefit in the addition of CT to the fractionated regimen, the side effects of CT significantly affected the quality of life and survival of the animals. Hence, further studies with a range of fractionated does with OER correction and equivalent BED on *in-vivo* models as well as fine-tuning of the chemoRT regimen might be necessary to ascertain the exact dose for translating this to the clinics. Although previous studies have found hypofractionation schemes for re-RT in NPC patients to be generally safe, effective and timesaving (36–38), the number of patients treated with this technique is too small make any definite conclusions.

Intensity modulated radiotherapy therapy (IMRT), together with non-invasive ^18^F-fluoromisonidazole (^18^F-MISO) hypoxia imaging, has made dose escalation to hypoxic sub-volumes technically possible. However, achieving this with precision, without affecting the organs at risk remains challenging (39, 40). This has sparked interest in proton therapy that could target high therapeutic radiation dose to the tumor with minimal exit dose (41, 42). Many institutions currently perform IMRT in combination with intensity modulated proton therapy (IMPT) for NPC (43–46), with promising local tumor control and reduction in side effects. Development of dose painting algorithm for dose escalation to hypoxic sub-volumes using combination IMRT and IMPT, could benefit from highly conformal and precise treatment delivery, potentially making this approach a paradigm shift in the re-treatment of recurrent NPC patients.

Taken together, this work highlights the development and characterization of patient derived 3D models of NPC that closely mimics cell-cell interactions, cellular heterogeneity, hypoxia, and radio-resistance. Hence, these models represent a straightforward, yet attractive technology that could complement *in-vivo* studies for better understanding of the underlying mechanism involved in tissue damage/repair, regeneration and response to therapy. However, the absence of human tumor microenvironment in these models is an inevitable drawback, which to an extend can be overcome by co-culture of the organoids with human immune cells and cancer associated fibroblasts. We further utilized the 3D models together with simple dose-survival data analytic techniques to yield quantitative readouts that defines the inherent radiobiological characteristic of radiosensitive normoxic and radioresistant hypoxic NPC. With combined experimental data, we conclude that hypoxic NPC require a large bolus dose or 1.4 times of the fractionated dose that is effective against normoxic cells in order to compensate for oxygen shortage. Further clinical results should be obtained in order to confirm its usefulness and translational value. In conclusion, this study could be a game changer in the way such models are utilized for optimization of radiation dose and our findings may have profound implications on how radiation treatments are planned in future, especially for re-irradiation of recurrent NPC.

## Data Availability Statement

The datasets presented in this study can be found in online repositories. The names of the repository/repositories and accession number(s) can be found below: NCBI Gene Expression Omnibus (https://www.ncbi.nlm.nih.gov/geo/query/acc.cgi?acc=GSE164544).

## Ethics Statement

Patient specimen collection and experimental use were approved by the institutional review board of National Healthcare Group (DSRB Reference: 2015/00098-SRF0004). The patients/participants provided their written informed consent to participate in this study. All animal care and experimental procedures were approved by the Institutional Animal Care and Use Committee, A*Star Research Entities, Singapore.

## Author Contributions

MT and SL conceived and designed the experiments. CL provided patient samples. SL performed the experiments. JS helped with establishment of PDX animal model. JM, MHL, and JW helped with RNA sequencing data analysis. ML, SY, DY, and SD analyzed dose-survival data. SL wrote the manuscript and performed literature, with the help of ML. All authors contributed to the article and approved the submitted version.

## Funding

This work was supported by Institute of Bioengineering and Nanotechnology (Biomedical Research Council, Agency for Science, Technology and Research (A*Star), Singapore).

## Conflict of Interest

ML, SY, DY, and SD were employed by the Proton Therapy Centre Pte. Ltd.

The remaining authors declare that the research was conducted in the absence of any commercial or financial relationships that could be construed as a potential conflict of interest.
