# Supplementary material for: Patient-Derived Nasopharyngeal Cancer Organoids for Disease Modeling and Radiation Dose Optimization
Source: Front Oncol. 2021 Feb 23;11:622244. doi: 10.3389/fonc.2021.622244 (PMC7959730; doi:10.3389/fonc.2021.622244)
Supplement: Supplementary file 1 [file DataSheet_1.docx]

**Supplementary Figures:**

**
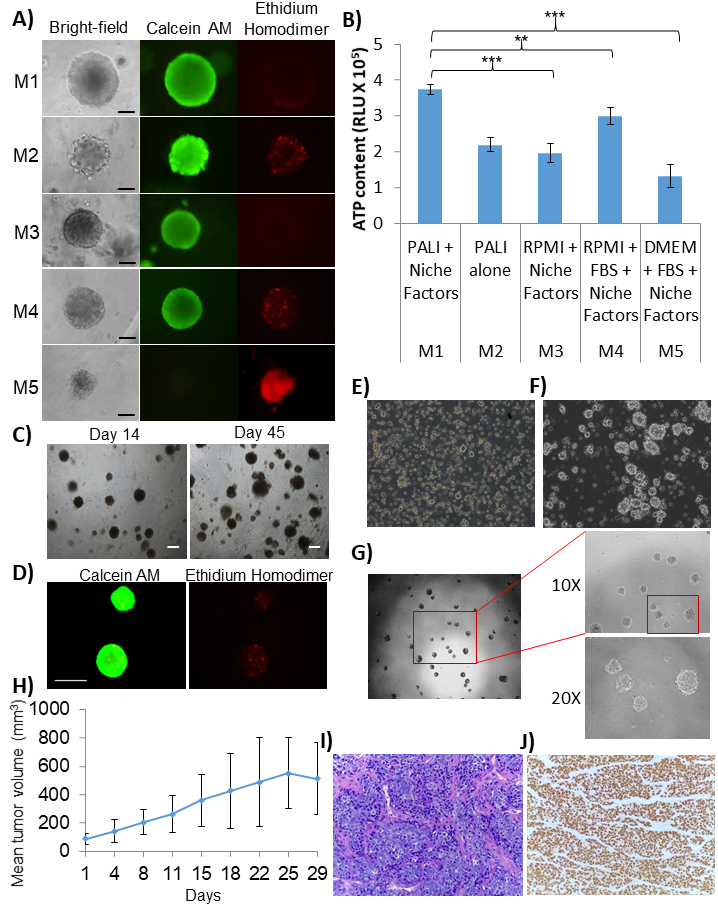
**

**Supplementary figure 1:** **Optimization of culture conditions.** (A) Live-dead assay of organoid culture at day 12 in different media (M1: PALI + niche factors (NF), M2: PALI alone, M3: RPMI + NF, M4: RPMI + 10% FBS + NF, M5: DMEM + 10% FBS + NF), Magnification 200X, scale bar 50 µm (B) End-point assay (Cell Titer Glo™) assay to assess the ATP content of organoids grown in different media without and with NF to optimize growth media. (C) Long term culture of organoids and growth patterns up to Day 45, Magnification 100X, scale bar 100 µm. (D) Live-dead assay of organoids at Day 45 (left panel, live = green, right panel red = dead), Magnification 200X, scale bar 100µm. (E) Morphology of single cell fraction (SCF) on day 0, right after its isolation during organoid culture. (F) Morphology of the self-assembled organoids formed from SCF on day 2. (G) Morphology of self-assembled organoids formed from SCF after encapsulation in geltrex, on day 3. (H) Tumorigenicity of NPC organoids in NSG mice, data shown are mean ± SD, n = 5. (I) H&E staining and (J) EBER ISH staining of tumor tissue formed from subcutaneous injection of organoids grown as suspension culture.


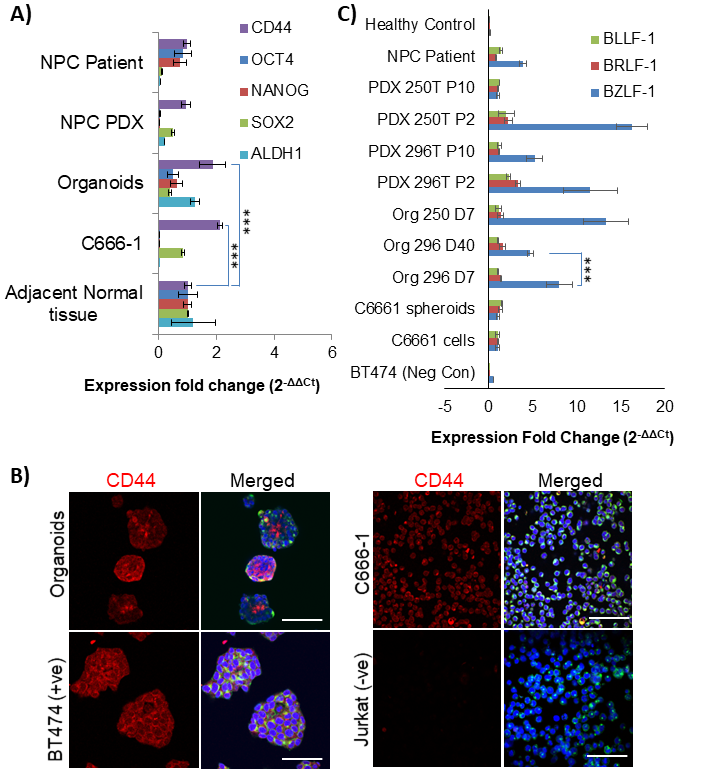


**Supplementary figure 2: Characterization of established organoids.** (A) Expression of various stem cell factor genes quantified by real-time PCR normalized to adjacent normal tissue, data shown are mean ± SD, n = 3. (B) Immunofluorescent staining of various samples with anti-CD44 (red), panel on the left are merged images with blue staining (Hoechst 33342) representing nucleus and green staining (WGA-488) representing cytoplasm, Magnification 200X, Scale bar 200 µm. (C) Expression of early (BZLF-1 and BRLF-1) and late (BLLF-1) genes quantified by real-time PCR normalized to expression of C666-1 cells. Data shown are mean ± SD, n = 3.


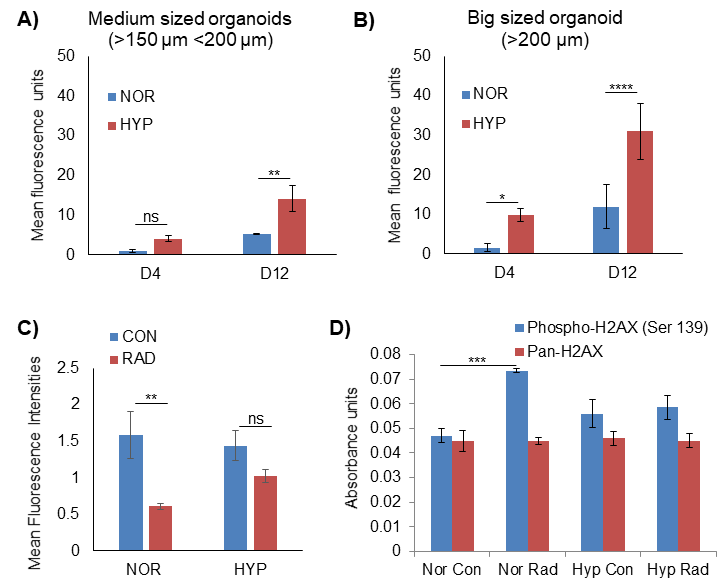


**Supplementary figure 3: Hypoxic region within organoids and molecular effects of RT on organoids.** (A) Quantification of GHR fluorescence of both normoxic and hypoxic organoids of medium size - >150 µm but <200 µm (B) and big size - >200 µm by image analysis using ImageJ software, data shown are mean ± SD, n = 5. ns = not significant, *p < 0.05, **p < 0.01 and ****P < 0.0001. (C) Quantification of Ki67 immunofluorescence staining in normoxic and hypoxic organoids without and with RT by image analysis, data shown are mean ± SD, n = 5, **p = 0.0012. (D) Quantification of pan-H2AX and phosphorylated-γH2AX in normoxic and hypoxic organoids without and with RT, data shown are mean ± SD, n = 3, ***P <0.01.
