# Supplementary material for: Patient-Derived Nasopharyngeal Cancer Organoids for Disease Modeling and Radiation Dose Optimization
Source: Front Oncol. 2021 Feb 23;11:622244. doi: 10.3389/fonc.2021.622244 (PMC7959730; doi:10.3389/fonc.2021.622244)
Supplement: Supplementary file 2 [file DataSheet_2.docx]

**Supplementary materials and methods:**

**PDX passaging, slow freezing, storage of PDX tumors and re-implantation of PDX tumors**

When the PDX tumors reached 1200-1500 mm^3^, the tissues were harvested. One part was used to re-implant in 5 new NSG mice (passaging) and the other part of the tumor tissue was cut into about 8 mm pieces and placed in cryovials with 1 ml RPMI 1640 media with 10% fetal bovine serum (FBS) and 10% DMSO. The vials were placed in a freezing container and transferred to -80°C freezer overnight. The following day vials containing the slow-frozen samples were transferred to liquid nitrogen (vapour phase) for long term storage.

To re-implant the slow frozen samples in mice, the frozen vials were quickly thawed in a 37°C water bath. The tissue samples were transferred to fresh 9 ml RPMI 1640 media with HEPES and L-Glutamine, 5X antibiotic/antimycotic and 5 µg/ml metronidazole. After dipping the tissue in an antibiotic cocktail containing 1:1 mix of Enrofloxacin (50 mg/ml) and Metronidazole (5 mg/ml) for about 10 min, it was triturated and mixed with Geltrex before subcutaneous implantation in mice.

**Harvesting organoids grown in Geltrex or suspension cultures**

Organoid cultures were established from PDX tissues each time and were not sub-cultured / passaged. Organoids grown in geltrex were incubated with 50 µl of 5 U/ml Dispase (Stemcell Technologies) and the plate was incubated at 37°C for 45 min to digest the gel. The organoids were then resuspended in Hanks' Balanced Salt Solution (HBSS), transferred to Eppendorf tube, washed at least 2 times before downstream applications such as imaging or DNA/RNA extraction or storage. For suspension cultures, organoids were simply passed through a 30 µm MACS Smart Strainer (Miltenyi Biotech), and the harvested organoids were resuspended in HBSS for further downstream applications.

**Histological characterization of patient and PDX tumor tissues and organoids**

Harvested tumor tissues (patients and mice) were fixed in 10% NBF, and were treated in an ascending series of ethanol and subsequently cleared with xylene. The processed tissues were then embedded in paraffin to prepare paraffin blocks. 5 µm thick sections were then cut using a microtome, placed on a slide and air-dried. Sections were dewaxed, rehydrated through descending ethanol series and then subjected to H&E staining. Following H&E staining, slides were subsequently dehydrated through ascending series of ethanol into xylene. Organoids were also fixed in 10% NBF for 20 min, and immobilized in 2% agarose gel block, that was processed and embedded in paraffin as mentioned above, before sectioning and H&E staining.

**DNA and RNA extraction and qPCR**

DNA extraction from cells, organoids and tissues was done using the DNeasy blood and tissue kit (Qiagen) following manufacturer’s instruction. Total RNA extraction from cells, organoids and tissues as well as formalin fixed paraffin embedded (FFPE) tissue was performed using the RNeasy mini kit (Qiagen) and RNeasy DSP FFPE Kit (Qiagen) (latter used only for 250T patient sample) respectively, following manufacturer’s recommendations. cDNA preparation was performed using High-Capacity cDNA Reverse Transcription Kit (Applied Biosystems) following manufacturer’s instructions. qPCR amplification was performed using SYBR™ Select Master Mix (Thermo Fisher Scientific) on a ViiA7 Real-time PCR system (Thermo Fisher Scientific) using the default qPCR conditions: Hold stage : 95°C for 10 min , PCR stage: 95°C for 15 sec and 60°C for 1 min for 40 cycles and Melt curve stage: 95°C for 15 sec –

| Gene | Strand | Primer sequence | Product size |
| --- | --- | --- | --- |
| GAPDH | Forward | 5’ - AGGGTCCCCGTCCTTGACTC- 3’ | **87 bp** |
|  | Reverse | 5’ - GATTGGCCCGATGGGAGGTG- 3’ |  |
| β-ACTIN | Forward | 5’ - GTCTTCCCCTCCATCGTG - 3’ | **113 bp** |
|  | Reverse | 5’ - AGGGTGAGGATGCCTCTCTT - 3’ |  |
| LMP2A | Forward | 5’ - AGCTGTAACTGTGGTTTCCATGAC - 3’ | **70 bp** |
|  | Reverse | 5’ - GCCCCCTGGCGAAGAG - 3’ |  |
| LMP1 | Forward | 5’ - CAGTCAGGCAAGCCTATGA - 3’ | **109 bp** |
|  | Reverse | 5’ - CTGGTTCCGGTGGAGATGA - 3’ |  |
| EBNA1 | Forward | 5’ - CCTACAGGGTGGAAAAATGGC - 3’ | **68 bp** |
|  | Reverse | 5’ - TCATCATCATCCGGGTCTCC - 3’ |  |
| BARF-1 | Forward | 5’ - AGGTCACCAAGCAGGAACAC - 3’ | **108 bp** |
|  | Reverse | 5’ - CGGTGCATGTCACAGTAAGG - 3’ |  |
| EBER | Forward | 5’ - AGGACCTACGCTGCCCTAGA - 3’ | **167 bp** |
|  | Reverse | 5’ - AAAACATGCGGACCACCAGC - 3’ |  |
| HIF-1a | Forward | 5’-CAGAGCAGGAAAAGGAGTCA-3’ | **232 bp** |
|  | Reverse | 5’-AGTAGCTGCATGATCGTCTG-3’ |  |
| OCT-4 | Forward | 5’ - TGGAGAAGGAGAAGCTGGAGCAAAA - 3’ | **186 bp** |
|  | Reverse | 5’ - GGCAGATGGTCGTTTGGCTGAATA - 3’ |  |
| NANOG | Forward | 5’ - GATTTGTGGGCCTGAAGAAA - 3’ | **155 bp** |
|  | Reverse | 5’ - AAGTGGGTTGTTTGCCTTTG - 3’ |  |
| CD44 | Forward | 5’ - TCAGAGGAGTAGGAGAGAGGAAAC - 3’ | **199 bp** |
|  | Reverse | 5’ - GAAAAGTCAAAGTAACAATAACAGTGG - 3’ |  |
| SOX-2 | Forward | 5’ - TACAGCATGTCCTACTCGCAG - 3’ | **110 bp** |
|  | Reverse | 5’ - GAGGAAGAGGTAACCACAGGG - 3’ |  |
| ALDH-1 | Forward | 5’ - TCCTGGTTATGGGCCTACAG - 3’ | **238 bp** |
|  | Reverse | 5’ - CTGGCCCTGGTGGTAGAATA - 3’ |  |
| BZLF1 | Forward | 5’ - AACTCCATGTCCTTCCAACG - 3’ | **91 bp** |
|  | Reverse | 5’ - GTGGGGGAATATGGGTCTCT - 3’ |  |
| BRLF1 | Forward | 5’ - GCTCAGGTCCATCTGTCCAC - 3’ | **68 bp** |
|  | Reverse | 5’ - GGGAGATGGCTGACACTGTT- 3’ |  |
| BLLF1 | Forward | 5’ – CATCTACAGATTCCAGGCTTACTTG - 3’ | **60 bp** |
|  | Reverse | 5’ - AGCTTCCAATTAACGTCACCA |  |

60°C for 1 min – 95°C for 15 sec. All samples were run in triplicate on 96 well PCR microplate. Gene expression quantiﬁcation was performed using Quantstudio™ Real-time PCR System (Thermo Fisher Scientific). Positively and negatively regulated genes were selected following a P<0.05 and an expression level of 1, respectively. All primers used are listed in the table below.

**EBER in situ hybridisation (EBER-ISH)**

EBER-ISH was performed using BOND Ready-to-use ISH EBER Probe from (Leica Biosystems) following manufacturer’s protocol. For detection BOND Polymer Refine Detection kit (Leica Biosystems) was used in accordance with the manufacturer’s recommendations.

**Immunofluorescent staining of cells and organoids**

Isolated organoids were fixed in 10% NBF for 20 min at RT and washed three times in PBS. Following fixation, organoids were subsequently permeabilized with 0.1% Triton X-100 for 15 min at RT and blocked with 5% bovine serum albumin in PBS for 1 h at RT. The permeabilized organoids were incubated with primary antibodies (rat anti-human ITGA6: clone NKI-GoH3; mouse anti-human CK20: clone Ks 20.8) at 1:50 dilution and incubated overnight at 4°C. Organoids were washed three times in 0.1% Tween 20 and incubated in secondary antibodies (goat anti-rat IgG-conjugated with AF488 and goat anti-mouse IgG2a-conjugated with AF488 respectively) at 1:400 dilution and incubated overnight at 4°C. For EpCAM staining a mouse monoclonal anti-EpCam antibody (Clone 9C4) conjugated to conjugated to Alexa Fluor® 488 was used at a concentration of 1:50. All antibodies were diluted in PBS with 5% BSA and 0.1% Tween 20. Organoids were counterstained with 5 µg/mL Hoechst 33342 for 10 min. Samples were washed 2 more times with HBSS before mounting on 8 well chambered coverglass slide.

**H2AX- ELISA and γ-H2AX Imaging**

To measure phosphorylated-H2AX in organoids that were and were not subjected to radiation, Human Phospho-H2AX (S139) and Total H2AX ELISA Kit (RayBio®), following manufacturer’s protocol. For γ-H2AX immunofluorescence imaging, cells and organoids were fixed with 10% NBF for 10 min. Permeabilization was performed by treating the cells and organoids with 0.1% Triton X-100 for 20 min. Blocking was done using 5% BSA with 0.1% Tween 20 in for 2 h. Anti-H2AX (pS139) Alexa 647-N1-431 antibody (BD), directed against γ-H2AX was prepared at 1:10 dilution and incubated overnight at 4°C. Following two washes (centrifugation at 1500 rpm at RT) with 0.1% Tween 20 the cells were counterstained with 5µg/mL AF488- Wheat Germ Aglutinin and 5 µg/mL Hoechst 33342 Hoechst for 10 min. Wash the samples 2 more times with HBSS before mounting on 8-well chambered cover glass slide.

**Microscopy**

Bright-field images were acquired using Olympus IX51 microscope. Conventional fluorescence images were obtained using an inverted Olympus IX-70 epifluorescence microscope. An inverted Olympus FV-1000 confocal laser scanning microscope was utilized to acquire immunofluorescence images (A*STAR, Singapore). Laser power, gain and pinhole levels were kept constant across the same experiment.

**Hypoxia measurement: Organoids and tissues**

Briefly, organoids cultured in 1% hypoxic and 21% normoxic conditions at day 4 and day 12 in geltrex were treated with 10 µM solution of Image-iT™ Green Hypoxia Reagent (GHR) (Invitrogen™) prepared according to manufacturer’s protocol and incubated at respective conditions at 37°C for 3 hours. After removing the dye, the gel was then digested by adding 50 µl of 5 U/ml Dispase and the plate was incubated at 37°C for 30-45 min. The organoids were then harvested by centrifugation at 1500 rpm for 5 min at RT, and the pellet was transferred to 8-well chambered cover glass slide (µ-Slide 8 Well, ibidi®) for live imaging by confocal laser scanning microscope.

For *in vivo* hypoxic imaging of the tumors, we used Hypoxyprobe Plus Kit from Hypoxyprobe Inc, USA according to manufacturer’s instructions with slight modification. Briefly, mice were given intravenous administration of 60 mg/kg pimonidazole HCl, which is reductively activated in hypoxic cells and form stable adducts with thiol groups. 90 min after the injection, mice were sacrificed and tumors were harvested and immediately fixed in 10% NBF. The tissues were then processed for IHC as mentioned elsewhere. The 5 µm sections were deparaffinized and subjected to antigen retrieval using Bond™ epitope retrieval solution for 40 min at 100°C followed by washing the slides 4 times with 1X Bond™ wash solution. Endogenous peroxidase blocking was done for 30 min in 3 - 4% (v/v) hydrogen peroxide solution followed by 5 times washing with 1X Bond™ wash solution. The sections were then incubated with 10% goat serum for 30 min at RT followed by incubation with 1:50 FITC-MAb1 (Hypoxyprobe Inc) in Bond™ antibody diluent for 60 min. Following washing step, the sections are incubated with rabbit anti-FITC secondary antibody at 1:50 dilution for 30 min. After washing the slides another 5 times, Bond™ Mixed DAB Refine is applied to each slide for 7 min, following which the slides are rinsed with deionised water, counterstained with hematoxylin for 5 min. Rinsed again with deionised water followed by 1X Bond™ wash solution, before dehydrating and mounting.

**Combined hypoxia and Ki67 staining of organoids**

Organoids in suspension culture grown 1% hypoxic and 21% normoxic conditions treated with a single dose of radiation at 5 Gy. 2 days after treatment the organoids were harvested, washed 2 times and stained with 10 µM solution of Image-iT™ GHR (Invitrogen™) prepared according to manufacturer’s protocol and incubated at 37°C for 5 hours. Organoids were subsequently washed 2 times in HBSS, and fixed with 10% NBF for 15 mins. Fixing, might reduce the intensity of GHR to some extend. The organoids were then permeabilized with 0.1% Triton X-100 for 15 min at RT and blocked with 5% bovine serum albumin in PBS for 1 h at RT and subsequently incubated with Anti-Ki-67 (Clone –SP6, 1:200, GeneTex) overnight at 4 °C. Following washing steps, the organoids were incubated with Goat anti-Rabbit IgG conjugated with Texas red (sc-3842 Santacruz, 1:400) overnight at 4 °C. Organoids were then counterstained with 5µg/mL Hoechst 33342 for 10 min and washed 2 more times with HBSS before mounting on 8 well chambered coverglass slide (ibidi µ-Slide).

**Immunohistochemical Ki67 staining of tumor tissues**

Tumor tissues were harvested and placed in a neutral buffered formalin overnight, processed in a tissue processor and embedded in paraffin wax. Paraffin-embedded tissues were cut into 4 μm thick sections and deparaffinized, cleared, and rehydrated in graded ethanol concentrations. Antigen retrieval was performed in 10mM citrate buffer (pH 6.0) for 20 min followed by cooling at RT for 20 min. Sections were pre-treated with peroxidase blocking buffer (3% H2O2 in methanol) for 20 min at RT. Blocking of non-specific binding was performed using 5% BSA for 1 h at RT. Anti-Ki-67 (Clone –SP6, 1:100, GeneTex) was incubated overnight at 4 °C. The sections were washed and incubated with peroxidase‑labelled polymer conjugated to goat anti‑rabbit immunoglobulin (Dako REAL™ EnVision™ Detection System, Peroxidase/DAB+ Rabbit/Mouse; Dako) at room temperature for 30 minutes. Subsequently, the sections were treated with Dako REAL DAB and Chromogen, and incubated for 5 minutes. The sections were then counterstained with hematoxylin, dehydrated and mounted with mounting medium. The bright field images were then analysed using ImageJ software using the color thresholding employing the Threshold_Colour plugin. At least 3 separate fields from each tumor tissue were used in image processing and quantification of the Ki67 indices per group. The fraction of the stained area was then calculated.

***In-vitro* radiation treatment: Single vs fractionated**

For hypo- vs hyper-fractionation experiments, the plates were irradiated with 1 x 20 Gy and 4 x 5 Gy dose respectively.

**References:**

[gProfiler] <https://biit.cs.ut.ee/gprofiler/gprofiler_nar_2019.pdf>

[xenome] <https://academic.oup.com/bioinformatics/article/28/12/i172/269972>

[STAR] <https://academic.oup.com/bioinformatics/article/29/1/15/272537>

[RSEM] <https://bmcbioinformatics.biomedcentral.com/articles/10.1186/1471-2105-12-323>

[DESeq2] <https://genomebiology.biomedcentral.com/articles/10.1186/s13059-014-0550-8>
